# Supplementary material for: The Crossregulation Triggered by Bacillus Strains Is Strain-Specific and Improves Adaptation to Biotic and Abiotic Stress in Arabidopsis
Source: Plants (Basel). 2024 Dec 20;13(24):3565. doi: 10.3390/plants13243565 (PMC11677973; doi:10.3390/plants13243565)
Supplement: Supplementary file 1 [file plants-13-03565-s001.zip › MATERIAL SUP/Melting curveS.pdf]

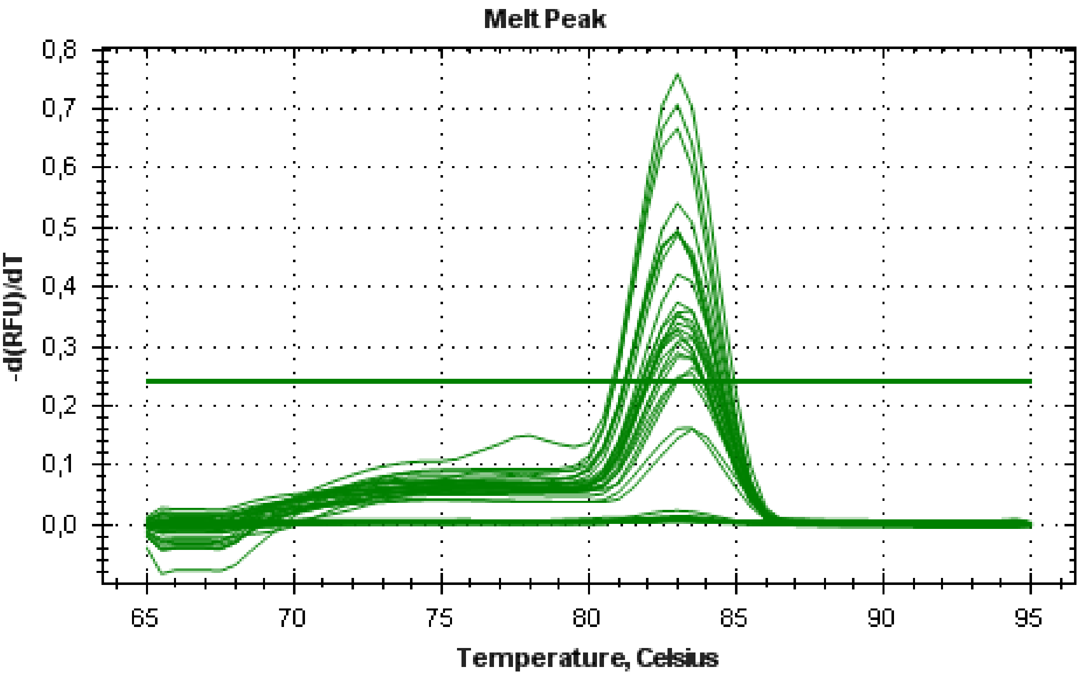

Figure S1a. Melting curve LOX2

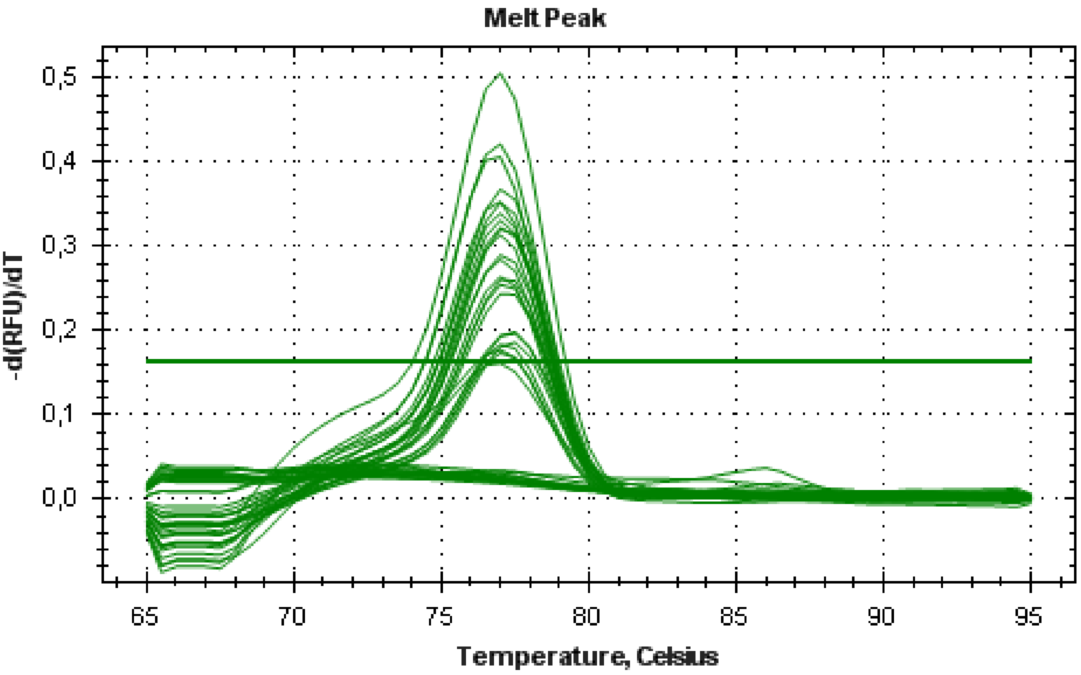

Figure S1b. Melting curve NPR1

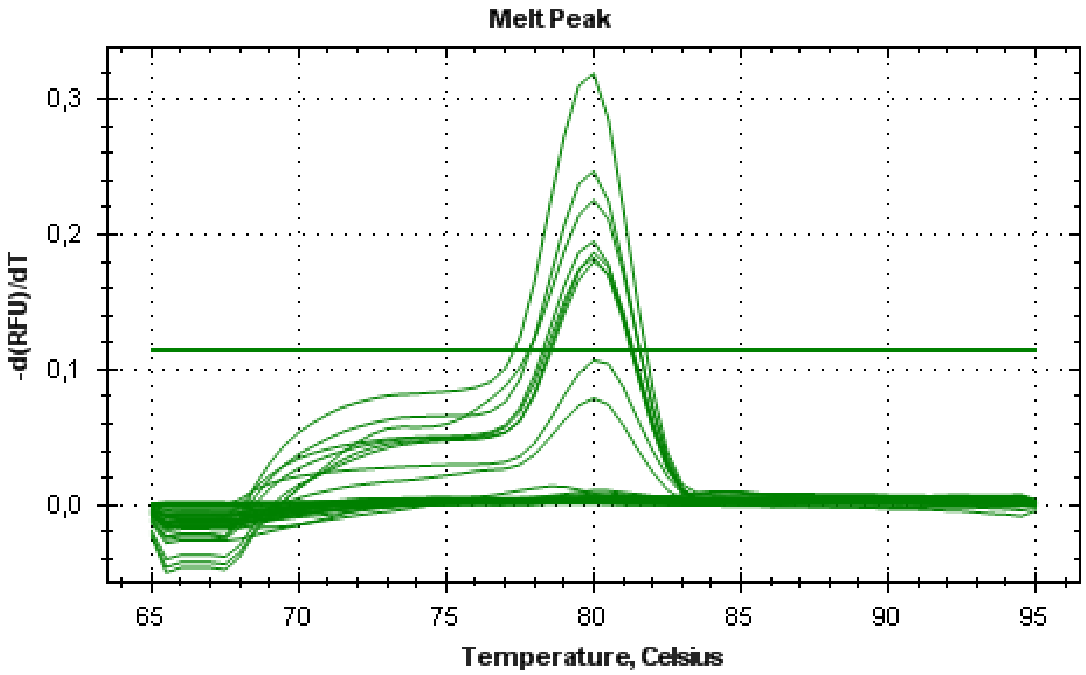

Figure S1c. Melting curve PDF1

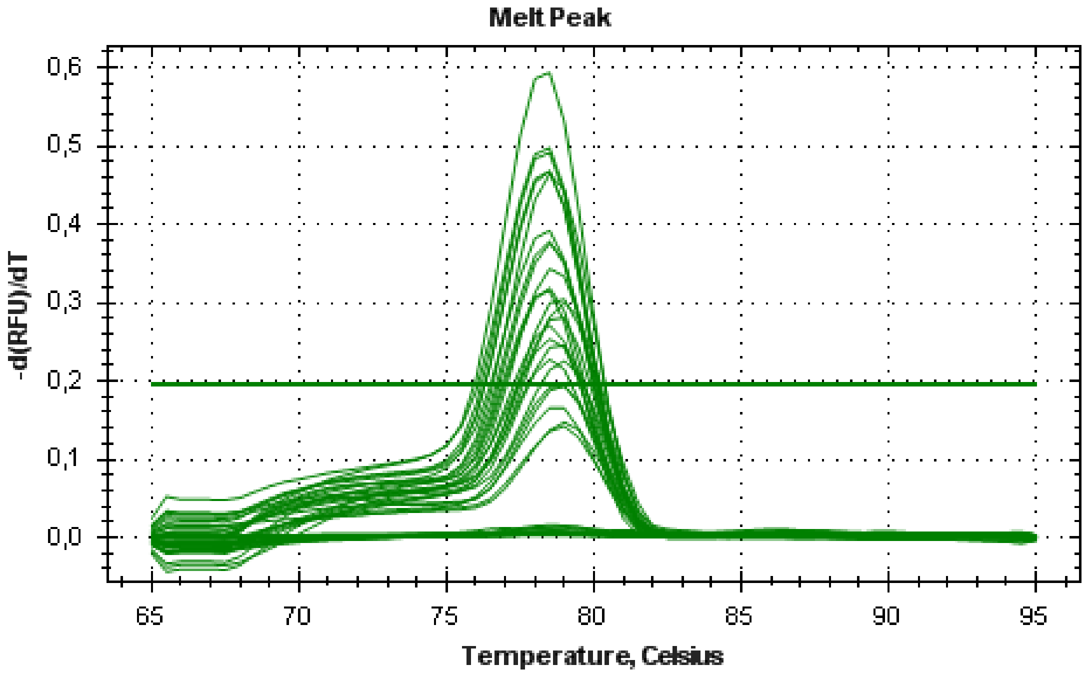

Figure S1d. Melting curve PR1

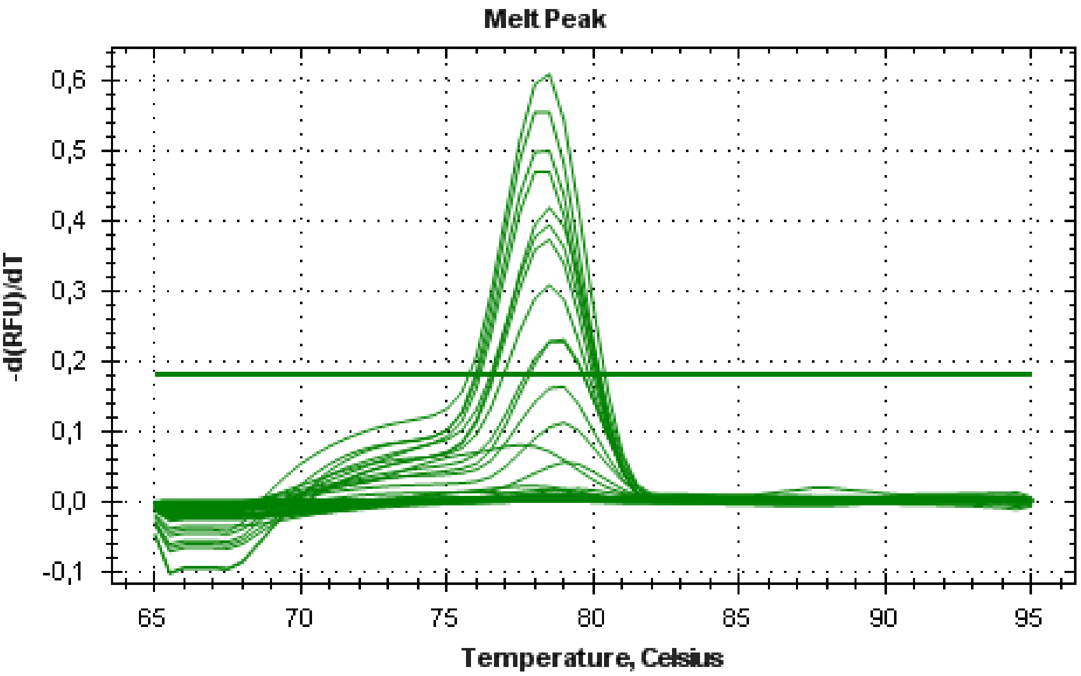

Figure S1e. Melting curve SAND
